# CSF biomarkers in patients with epilepsy in Alzheimer’s disease: a nation-wide study

**DOI:** 10.1093/braincomms/fcac210

**Published:** 2022-08-17

**Authors:** Rakesh Kumar Banote, Samuel Håkansson, Henrik Zetterberg, Johan Zelano

**Affiliations:** Department of Neurology, Sahlgrenska University Hospital, Gothenburg 41345, Sweden; Department of Clinical Neuroscience, Sahlgrenska Academy, University of Gothenburg, Sweden; Wallenberg Centre for Molecular and Translational Medicine, University of Gothenburg, Sweden; Department of Neurology, Sahlgrenska University Hospital, Gothenburg 41345, Sweden; Department of Clinical Neuroscience, Sahlgrenska Academy, University of Gothenburg, Sweden; Wallenberg Centre for Molecular and Translational Medicine, University of Gothenburg, Sweden; Department of Psychiatry and Neurochemistry, Institute of Neuroscience and Physiology, the Sahlgrenska Academy at the University of Gothenburg, Mölndal 43180, Sweden; Clinical Neurochemistry Laboratory, Sahlgrenska University Hospital, Mölndal 43180, Sweden; Department of Neurodegenerative Disease, UCL Institute of Neurology, Queen Square, London WC1E 6BT, UK; UK Dementia Research Institute at UCL, London WC1E 6BT, UK; Hong Kong Center for Neurodegenerative Diseases, Clear Water Bay, Hong Kong, China; Department of Neurology, Sahlgrenska University Hospital, Gothenburg 41345, Sweden; Department of Clinical Neuroscience, Sahlgrenska Academy, University of Gothenburg, Sweden; Wallenberg Centre for Molecular and Translational Medicine, University of Gothenburg, Sweden

**Keywords:** Alzheimer’s disease, epilepsy, cerebrospinal fluid, biomarkers

## Abstract

Alzheimer’s disease is the most common neurodegenerative dementia. A subset of Alzheimer’s disease patients develop epilepsy. The risk is higher in young-onset Alzheimer’s disease, but pathophysiological mechanisms remain elusive. The purpose of this study was to assess biomarkers reflecting neurodegeneration in Alzheimer’s disease patients with and without epilepsy. By cross-referencing the largest national laboratory database with Swedish National Patient Register, we could identify CSF biomarker results from 17901 Alzheimer’s disease patients, and compare levels of neurofilament light, glial fibrillary acidic protein, total tau, phosphorylated tau and amyloid beta 42 in patients with (*n* = 851) and without epilepsy. The concentrations of total tau and phosphorylated tau were higher in Alzheimer’s disease patients with epilepsy than Alzheimer’s disease patients without epilepsy and amyloid beta 42 levels were significantly lower in Alzheimer’s disease patients with epilepsy. No differences in the levels of neurofilament light and glial fibrillary acidic protein were observed. Our study suggests that epilepsy is more common in Alzheimer’s disease patients with more pronounced Alzheimer’s pathology, as determined by the CSF biomarkers. Further studies are needed to investigate the biomarker potential of these CSF markers as predictors of epilepsy course or as indicators of epileptogenesis in Alzheimer’s disease.

## Teaser

Banote *et al*. studied CSF biomarker levels in Alzheimer disease with and without epilepsy. They found higher T-tau and P-tau and lower Aβ_42_ concentrations in Alzheimer disease patients with epilepsy compared with Alzheimer disease patients without epilepsy, suggesting that development of epilepsy is associated with a more pronounced neurodegenerative Alzheimer disease process.

## Graphical Abstract

**Graphical abstract:**
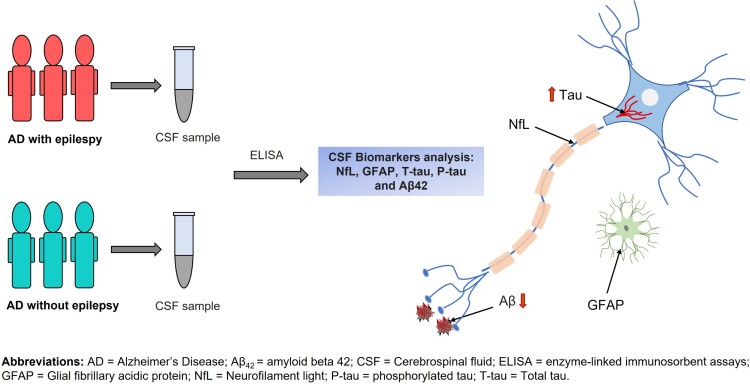

## Introduction

Alzheimer’s disease carries an increased risk of epilepsy.^1,2^ The pathophysiology remains elusive. Theoretically, Alzheimer’s disease-associated epilepsy could result from an individual predisposition to seizures triggered by neurodegeneration in vulnerable individuals, or from specific features of the neurodegenerative process. Studies of clinical characteristics seem to favour the latter explanation; young-onset and clinically severe Alzheimer’s disease are risk factors for epilepsy, and persons with Alzheimer’s disease and epilepsy may have higher cognitive decline and faster progression of symptoms.^3^

CSF reflect brain changes and quantification of CSF biomarkers is increasingly used in Alzheimer’s disease.^4,5^ Total tau (T-tau), phosphorylated tau (*P*-tau) and amyloid beta 42 (Aβ_42_) are key pathological biomarkers for the diagnosis and staging of Alzheimer’s disease.^6^ Neurofilament light (NfL) reflects axonal degeneration and injury.^7^ Glial fibrillary acidic protein (GFAP) reflects astroglial activation or blood–brain barrier dysfunction across a broad range of acute and chronic neurological diseases.^8^ Whether biomarker profiles differ in persons with or without Alzheimer’s disease-related epilepsy remains unknown.

In an attempt to elucidate whether epilepsy develops in patients with Alzheimer’s disease because of degenerative changes or individual predisposition, we asked if biochemical marker levels differed between Alzheimer’s disease patients with and without epilepsy. We used 20 years of laboratory data at Sahlgrenska University Hospital, the largest national provider of CSF biomarker analyses, and comprehensive national patient registers to identify 17901 individuals diagnosed with Alzheimer’s disease and compared CSF profiles of Alzheimer’s disease patients with and without epilepsy.

## Materials and methods

### Registers and study cohort

**Figure 1:**
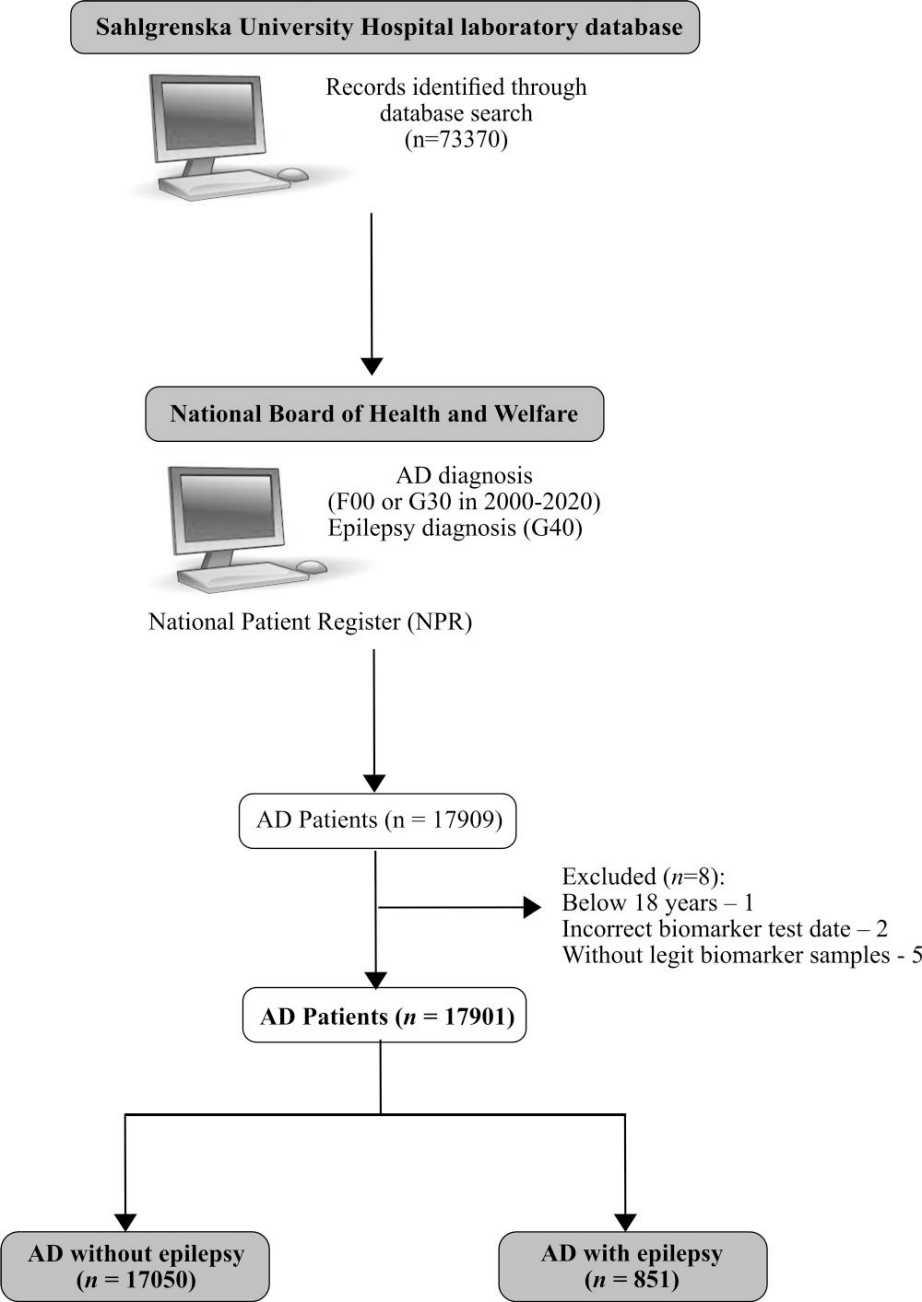
**Study flow chart describing cohort’s identification.** After database search for CSF tau in Sahlgrenska University Hospital, the data were sent to National Board of Health and Welfare to identify individuals with a diagnosis of Alzheimer’s disease in the National Patient Register (NPR). Diagnoses of Alzheimer’s disease and epilepsy were ascertained by identification of relevant ICD codes. The final study cohort included 17901 Alzheimer’s disease patients, of which 851 had epilepsy and 17050 patients were without epilepsy.

The Clinical Neurochemistry Laboratory at Sahlgrenska University Hospital was among the first in Sweden to analyze CSF biomarkers in Alzheimer’s disease and for many years the sole national provider of these analyses. The laboratory database was searched for all individuals with an entry for CSF tau (any form). The search identified 73370 individuals. For these individuals, we obtained all CSF analyses for brain injury markers. The data were sent to the National Board of Health and Welfare to identify individuals with a diagnosis of Alzheimer’s disease in the National Patient Register (NPR). NPR contains information on all inpatient hospital admissions since 1987 and hospital-based outpatient visits since 2001. Diagnoses of Alzheimer’s disease and epilepsy were ascertained by identification of relevant International Classification of Diseases, 10th version (ICD-10) criteria; code F00 or G30 for Alzheimer’s disease and code G40 for epilepsy. Comorbidities that could also cause epilepsy or affect biomarker levels were identified by the relevant ICD-10 codes: stroke (I60-I69), traumatic brain injury (S00-S09) and CNS neoplastic disease (C71, C793, D430, D32, D330). Information on anti-seizure medication (ASM) was obtained from the Drug Register, which contains information on all dispensed drugs in Sweden since 2005. The final study cohort included 17901 Alzheimer’s disease patients, of which 851 also had epilepsy (Fig. 1- Flow chart).

For the analysis of CSF biomarkers, we included all Alzheimer’s disease–epil patients with onset of epilepsy after Alzheimer’s disease diagnosis and age >55 at Alzheimer’s disease onset. Case ascertainment in this analysis was based solely on the diagnostic code. Age- and sex-matched controls were selected from Alzheimer’s disease patients without epilepsy. In one sensitivity analysis, individuals with epilepsy and controls with a diagnosis of trauma or stroke before the CSF test were excluded, since such CNS insults may alter biomarker levels. In an additional sensitivity analysis, patients with CNS neoplastic disease were excluded.

### Ethical approval

This study was approved by Swedish Ethical Review Authority (approval number 2020-05717). The National Board of Health and Welfare anonymized all data after linkage and before we had access to them. All handling of personal data was done in agreement with Swedish data protection laws.

### CSF biomarkers

The CSF samples were obtained by lumbar puncture according to standard procedures as described previously.^9^ Biomarkers were measured at the Clinical Neurochemistry Laboratory at Sahlgrenska University Hospital. CSF was analyzed continuously as part of routine clinical practice. The samples were analyzed using commercially available enzyme-linked immunosorbent assays (ELISA) to determine the levels of T-tau, Aβ42, *P*-tau (INNOTEST, Fujirebio, Ghent, Belgium), and NfL (UmanDiagnostics, Umeå, Sweden). GFAP levels were quantified using an in-house ELISA based on polyclonal antibodies.^10^ The biomarker measurements were performed by board-certified laboratory technicians who were blind to clinical data and used protocols accredited by the Swedish Board for Accreditation and Conformity Assessment.

### Statistical analysis

Biomarkers were compared in matched analyses where each Alzheimer’s disease patient with epilepsy was matched with a control (Alzheimer’s disease without epilepsy) with the same sex and closest age. If more than one sample of a biomarker was available for a patient, the sample taken closest to the date of Alzheimer’s disease onset was used for analysis. Subgroups analyzed were onset before or after age 65. The matching was then performed to match each Alzheimer’s disease patient with epilepsy with a control. In sensitivity analyses, patients with stroke or traumatic brain injury before a CSF test were removed (if for a patient, an earlier test was available before stroke or trauma, that test was used) and a separate analysis removed patients with CNS neoplastic disease before the CSF test. CSF biomarker levels were assessed using Student’s *t*-test. Levels were considered significantly regulated at *P* < 0.05. A standard indicator of statistical significance was used in the figures (ns *P* > 0.05, *P ≤ 0.05, **P ≤ 0.01, ***P ≤ 0.001, ****P ≤ 0.0001). Data were analyzed using IBM SPSS Statistics, version 26.0 for Windows and statistical analysis was performed using R software (version 4.0.2).

### Data availability

The data set for this study is protected by Swedish privacy laws and agreements between Sahlgrenska University Hospital and the register holder (National Board of Health and Welfare) and cannot be shared by the authors.

## Results

### Study cohort

**Figure 2:**
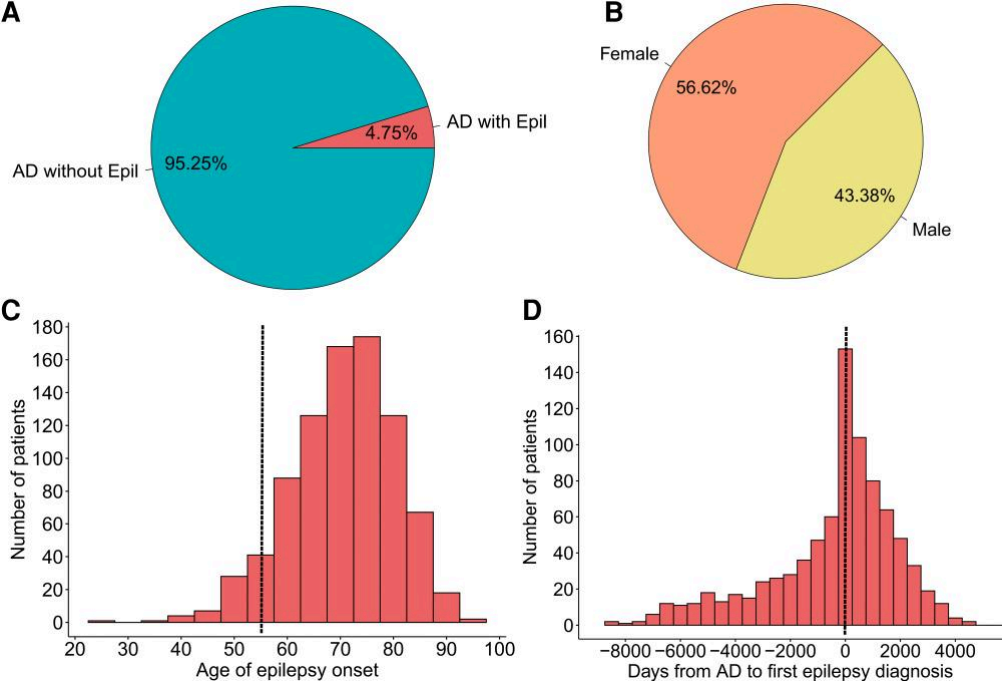
**The study cohort.** (**A**) Population of Alzheimer’s disease with and without epilepsy and (**B**) sex distribution. (**C**) Histogram showing age of epilepsy onset (patients >55 years of age to the right of dashed line were included) and (**D)** days from Alzheimer’s disease to epilepsy diagnosis (patients with epilepsy onset after Alzheimer’s disease, to the right of the dashed line were included).

The study cohort consisted of 17901 patients, where 851 (4.75%) Alzheimer’s disease patients had epilepsy and 17050 (95.25%) were without epilepsy (Fig. 2A). The demographic and clinical characteristics of the patients are shown in Table 1. The age and sex distributions were comparable between the groups (Fig. 2B, Table 1), but stroke (28%) and trauma (31%) were more common in Alzheimer’s disease patients with epilepsy compared with Alzheimer’s disease patients without epilepsy (Table 1). Epilepsy onset in Alzheimer’s disease was more common between 60 and 80 years of age (Fig. 2C), and the first epilepsy diagnosis was often close to Alzheimer’s disease diagnosis (Fig. 2D). The time of the CSF analysis in relation to the Alzheimer’s disease diagnosis was similar in patients with and without epilepsy (Table 1).

**Table 1:** Study cohort: description of demographics and clinical characteristics of the patients

	Alzheimer’s disease without Epil	Alzheimer’s disease with Epil
***N (%)***	17050 (95.25%)	851 (4.75%)
**Sex**		
Male	7358 (43.2%)	408 (47.9%)
Female	9692 (56.8%)	443 (52.1%)
**Age at export, mean ± SD**	74.10 ± 7.93	71.61 ± 9.25
**Epilepsy age (Age at Epi diagnosis)**		70.51 ± 9.87
**Age at CSF test, mean ± SD**	73.34 ± 7.77	71.18 ± 9.16
**Time from Alzheimer’s disease diagnosis to CSF test, mean ± SD**	–215.99 ± 600.53	–239.18 ± 647.98
**Deceased**	9018 (52.9%)	505 (59.3%)
**Comorbidities**		
Stroke	2309 (13.5%)	238 (28.0%)
Trauma	3449 (20.2%)	265 (31.1%)
**ASM**	2467 (14.5%)	750 (88.1%)

### CSF measures

**Figure 3:**
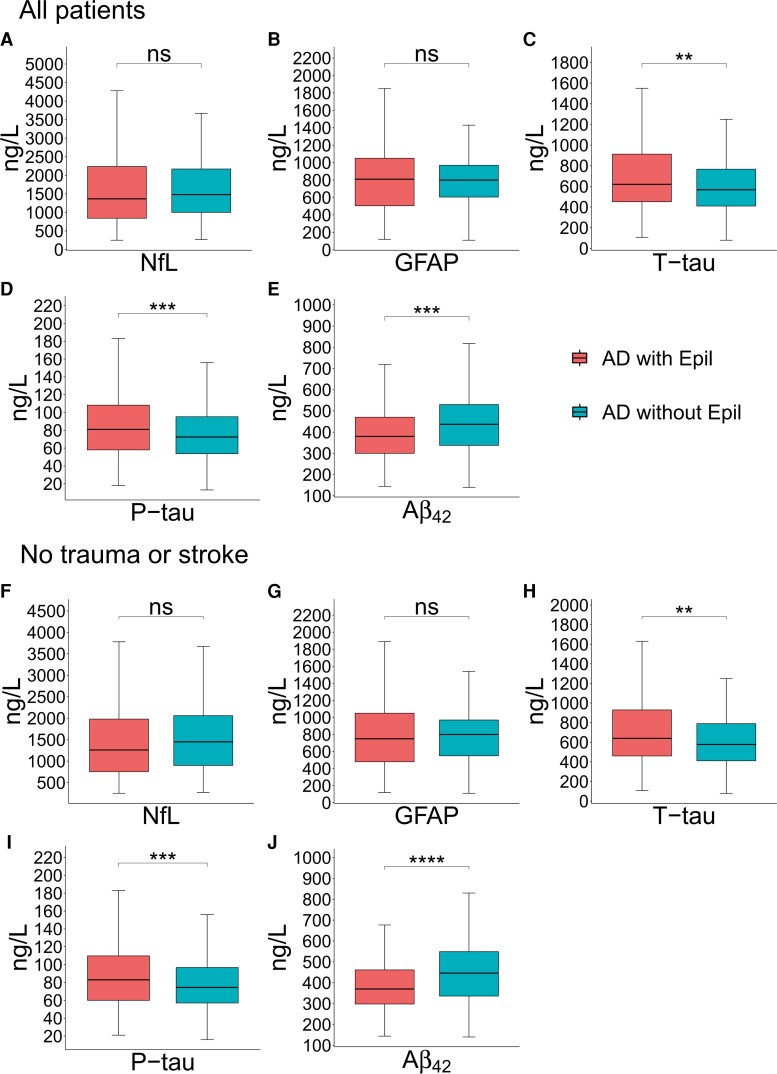
**Analysis of CSF biomarkers in Alzheimer’s disease patients with and without epilepsy (**A–E**)**. (NfL: *n* = 226, GFAP: *n* = 83, T-tau: *n* = 384, *P*-tau: *n* = 364, Aβ_42_: *n* = 364 per group). Boxes show the median, first and third quartile, and minimum and maximum value (excluding outliers). Student’s *t*-test T-Tau (*P* = 0.0019), *P*-Tau (*P* = 0.0002) and Aβ_42_ (*P* = 0.0002). Excluded patients who had stroke and trauma before CSF test (**F–J**). (NfL: *n* = 185, GFAP: *n* = 73, T-tau: *n* = 320, *P*-tau: *n* = 302, Aβ_42_: *n* = 302 per group). Boxes show the median, first and third quartile, and minimum and maximum value (excluding outliers). Student’s *t*-test T-tau (*P* = 0.0047), *P*-tau (*P* = 0.0004) and Aβ_42_ (*P* < 0.0001).

CSF biomarker levels were analyzed in Alzheimer’s disease–ep patients, and age- and sex-matched controls (NfL: *n* = 226, GFAP: *n* = 83, T-tau: *n* = 384, *P*-tau: *n* = 364, Aβ42: *n* = 364 per group). The concentrations of T-tau and *P*-tau were higher in Alzheimer’s disease patients with epilepsy (*P* = 0.0019 and *P* = 0.0002, respectively) compared with Alzheimer’s disease patients without epilepsy [median, min–max; T-tau: 620 (107–6940) ng/l versus 567.5 (79–2480) ng/l, *P*-tau: 81 (18–253) ng/l versus 72.5 (13–197) ng/l] (Fig. 3C and D). The Aβ_42_ levels were significantly lower (*P* = 0.0002) in Alzheimer’s disease patients with epilepsy [median, min–max; Aβ_42_: 380 (144–1550) ng/l versus 437 (140–1140) ng/l] (Fig. 3E). There were no significant differences in the levels of NfL and GFAP (Fig. 3A and B).

**Figure 4:**
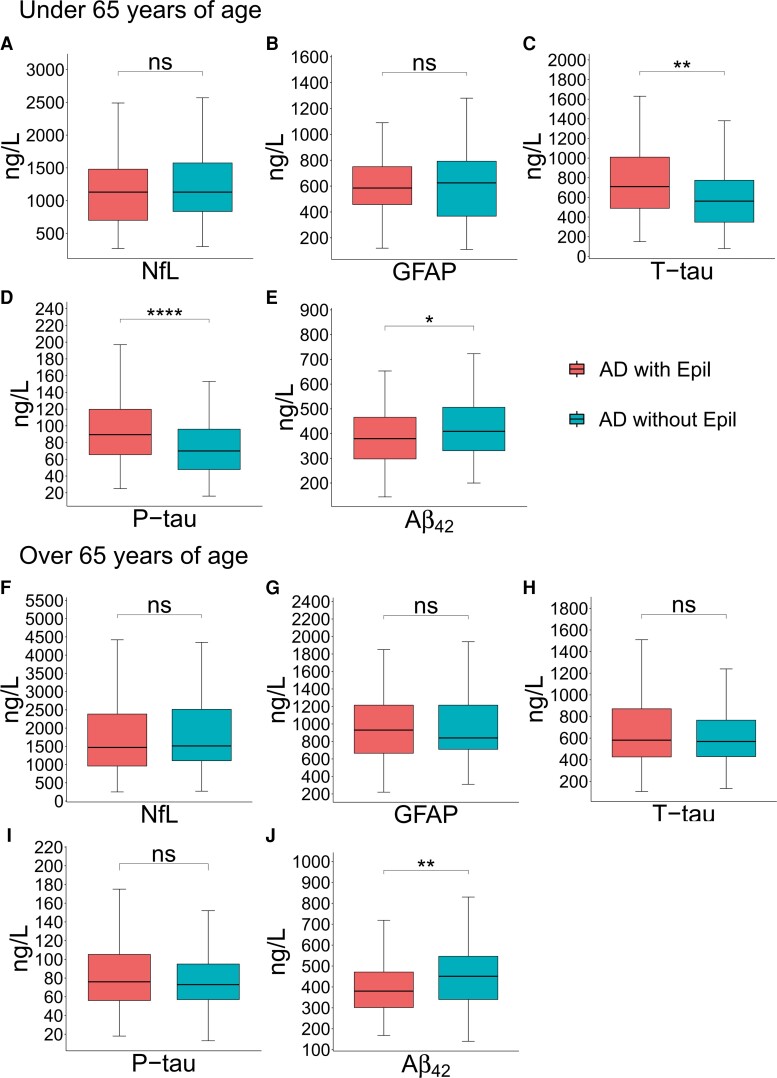
**Analysis of CSF biomarkers in Alzheimer’s disease patients with and without epilepsy aged younger than (A-E) and older than 65 years (F-J).** (Younger than 65 years of age: NfL: *n* = 59, GFAP: *n* = 28, T-tau: *n* = 115, *P*-tau: *n* = 108, Aβ_42_: *n* = 108 per group and older than 65 years of age: NfL: *n* = 167, GFAP: *n* = 55, T-tau: *n* = 269, *P*-tau: *n* = 256, Aβ_42_: *n* = 256 per group). Boxes show the median, first and third quartile and minimum and maximum value. Student’s *t*-test aged younger than 65 years: T-Tau (*P* = 0.0025), *P*-Tau (*P* < 0.0001) and Aβ_42_ (*P* = 0.0151) and older than 65 years: Aβ_42_ (*P* = 0.004).

In a sensitivity analysis, we excluded patients with other insults that could have affected CSF biomarker levels like stroke or traumatic brain injury before the CSF test (NfL: *n* = 185, GFAP: *n* = 73, T-tau: *n* = 320, *P*-tau: *n* = 302, Aβ_42_: *n* = 302 per group). This did not alter the results; levels of T-tau (*P* = 0.0047) and *P*-tau (*P* = 0.0004) were still higher in Alzheimer’s disease patients with epilepsy [median, min–max; T-tau: 640 (108–6940) ng/l versus 577.5 (79–2480) ng/l, *P*-tau: 83 (21–253) ng/l versus 74.5 (16–197) ng/l], and the levels of Aβ_42_ [*P* < 0.0001) were lower (median, min–max; Aβ_42_: 370 (144–1200) ng/l versus 446 (140–1450) ng/l] (Fig. 3H, I, and J). Similarly, we found no significant differences in concentrations of NfL and GFAP between the groups if patients with stroke or traumatic brain injury before the CSF were excluded (Fig. 3F and G). In an additional sensitivity analysis, we excluded patients with codes for CNS neoplastic disease, which did not alter the results (Supplementary Table 1). We also performed analysis on patients under and over 65 years of age (younger than 65 years of age: NfL: *n* = 59, GFAP: *n* = 28, T-tau: *n* = 115, *P*-tau: *n* = 108, Aβ_42_: *n* = 108 per group and older than 65 years of age: NfL: *n* = 167, GFAP: *n* = 55, T-tau: *n* = 269, *P*-tau: *n* = 256, Aβ_42_: *n* = 256 per group), where Aβ_42_ was significantly lower in both groups (younger than 65 years of age: *P* = 0.0151 and older than 65 years of age *P* = 0.004) (Fig. 4E and J) and T-tau and *P*-tau levels were significantly higher in the younger than 65 years of age group (*P* = 0.00245 and *P* < 0.0001, respectively) (Fig. 4C and D) [younger than 65 years of age: median, min–max; T-tau: 710 (150–6940) ng/l versus 562 (79–1870) ng/l, *P*-tau: 89.5 (25–253) ng/l versus 70 (16–189) ng/l, Aβ_42_: 379.5 (144–952) ng/l versus 409 (200–1140) ng/l and older than 65 years of age: median, min–max; T-tau: 581 (107–3400) ng/l versus 569 (134–2480) ng/l, *P*-tau: 76 (18–250) ng/l versus 73 (13–197) ng/l, Aβ_42_: 380 (168–1550) ng/l versus 451 (140–1100) ng/l].

## Discussion

In this study, we describe the profile of CSF biomarkers that reflect neurodegeneration and Alzheimer’s disease pathological processes in patients with and without epilepsy. This is the first and largest investigation of CSF biomarkers in this patient group, and the study offers insights into possible pathophysiological mechanisms of seizures in Alzheimer’s disease. We found epilepsy to be associated with increased levels of CSF T-tau and *P*-tau and decreased levels of Aβ_42_. These are key changes that are specifically associated with Alzheimer’s disease and not associated with other neurodegenerative dementias,^4^ which suggests that the development of epilepsy is linked to a more pronounced Alzheimer’s disease process. Put differently—patients with epilepsy in Alzheimer’s disease seem to have a more marked biochemical Alzheimer’s disease profile than patients who do not develop epilepsy.

The aggregation of hyperphosphorylated tau (also known as neurofibrillary tangles) in the cell body is a key pathological feature of Alzheimer’s disease. Increased phosphorylation and release of tau from neurons in CSF appears to reflect a neuronal response to Aβ deposition in Alzheimer’s disease.^11,12^ Tau is attracting increasing interest in both epilepsy and dementia research; higher levels of tau aggregation have been described in brain tissue from Alzheimer’s disease patients with seizures, perhaps reflecting greater damage to cortical neuronal networks or epileptogenesis in Alzheimer’s disease.^13–17^ Interestingly, tau aggregation is also described in non-Alzheimer’s disease epilepsy and is linked to both seizure frequency and cognitive decline.^17–19^ Our findings of higher levels of T-tau and *P*-tau in Alzheimer’s disease patients with epilepsy are in agreement with these observations, but whether the increased tau levels cause or reflect seizure activity remains to be determined.

Aβ is a secreted proteolytic cleavage product of the transmembrane amyloid precursor protein, and accumulation of Aβ_42_ into extracellular plaques in the brain is an early event in Alzheimer’s disease pathogenesis.^20^ Aggregation of Aβ_42_ in the brain parenchyma results in reduced concentration of the protein in CSF.^21^ Low CSF Aβ_42_ concentration is associated with late-onset epilepsy and subsequent development of Alzheimer’s disease,^22,23^ but differences between Alzheimer’s disease patients with and without epilepsy have to our knowledge not been reported previously.

In summary, we found more pronounced biochemical evidence of Alzheimer’s disease pathology in Alzheimer’s disease patients with epilepsy. In contrast, we found no differences in NfL and GFAP levels between Alzheimer’s disease patients with and without epilepsy. These proteins are used as general markers of neurodegeneration and astrocytic activation and are not Alzheimer’s disease -specific.^9,24^ The absence of an association of CSF NfL and GFAP with epilepsy in Alzheimer’s disease further underscores that epilepsy in Alzheimer’s disease is associated with Alzheimer’s disease pathology as such and not general neurodegenerative brain changes. A subgroup analysis of individuals younger versus older than 65 years of age showed similar significant changes in the Aβ_42_ levels, whereas T-tau and *P*-tau concentrations were significantly higher in the epilepsy compared with the non-epilepsy group only among younger individuals. This may be due to increased prevalence of subclinical Alzheimer’s disease pathology in older age groups, making Alzheimer’s disease biomarker results overall less informative in the elderly.^25,26^

One important factor to consider when interpreting the results of this study is the timing of the CSF analysis. Lumbar puncture is a standard component of a dementia workup, with memory problems being the main indication. In general, the lumbar puncture in our material was performed before the Alzheimer’s disease diagnosis was made. Since our analysis only included individuals who were diagnosed with epilepsy after the Alzheimer’s disease diagnosis, the selective difference detected in our material for tau and Aβ_42_ could indicate that epilepsy develops in patients with a more severe Alzheimer’s disease trajectory. It is well in line with clinical observations that seizures tend to develop in severe Alzheimer’s disease.^27^ Unfortunately, we did not have access to dementia severity for our cohort, but an interesting future study for increased patophysiological understanding could be to match cases with and without epilepsy of similar Alzheimer’s disease severity. Similarly, we did not have access to data on epilepsy severity and in future studies association between a a more pronounced Alzheimer’s disease CSF profile and seizure frequency would be very interesting. However, the correlation between CSF profile and PET of tau brain pathology is not absolute, and tau-PET seems superior to CSF analysis when it comes to analysing disease progression,^28^ so a multimodal approach including functional imaging would probably be of greatest value when trying to understand how Alzheimer’s disease pathology causes seizures. Regional distribution of Alzheimer’s disease pathology to particularly epileptogenic brain regions such as the temporal lobe could also be interestig to explore in imaging studies.

To our knowledge, the combination of data from the largest national analysis provider with national register data has resulted in the largest study so far of biochemical markers in Alzheimer’s disease, covering CSF analyses between 2000 and 2021. Apart from the study size, another advantage of our approach is the unbiased detection of an administrative epilepsy diagnosis. Drawbacks include our reliance on administrative data. Alzheimer’s disease and epilepsy diagnoses are sometimes erroneous, but more importantly seizures are not seldom overlooked in patients with dementia. In general, the PPV of a dementia diagnostic code in the NPR is high (>80%), but the PPV for Alzheimer’s disease specifically is lower (56%),^29^ so a subset of the patients with Alzheimer’s disease will have another reason for their dementia. However, given that our study population was examined with a lumbar puncture suggesting a dementia-interested center and that most patients received their Alzheimer’s disease diagnosis after the lumbar puncture, we suspect that the diagnostic accuracy is higher in our material. This is supported by the fact that the non-epilepsy group also had Aβ_42_ levels below normal and indicative of true Alzheimer’s disease.^30^ The PPV of a diagnosis of epilepsy is about 90%.^31^ Importantly, diagnostic errors in the presence of absence of epilepsy are unlikely to be systematic with regard to the biomarker levels. Another way of interpreting our findings is that presence of epilepsy makes it more likely that a person with an Alzheimer’s disease diagnosis will have CSF biomarker results with a pronounced Alzheimer’s disease profile.

In summary, we found a more pronounced Alzheimer’s disease biomarker profile concerning the levels of T-tau, *P*-tau and Aβ_42_ in Alzheimer’s disease patients with epilepsy. More studies are needed. In addition to elucidating the pathophysiological processes underlying Alzheimer’s disease, an interesting question is whether the biochemical profile can contribute to increased clinical awareness of seizures in Alzheimer’s disease. With the emergence of new drugs in dementia, a key question is also whether disease-modifying drugs can delay or prevent epileptogenesis.

## Supplementary Material

fcac210_Supplementary_Data

## Abbreviations

Aβ_42_ =: amyloid beta 42
GFAP =: glial fibrillary acidic protein
ICD =: International Classification of Diseases
NfL =: neurofilament light
NPR =: National Patient Register
*P*-tau =: phosphorylated tau
T-tau =: total tau
