# Supplementary material for: CSF biomarkers in patients with epilepsy in Alzheimer’s disease: a nation-wide study
Source: Brain Commun. 2022 Aug 17;4(4):fcac210. doi: 10.1093/braincomms/fcac210 (PMC9419062; doi:10.1093/braincomms/fcac210)
Supplement: fcac210_Supplementary_Data [file fcac210_supplementary_data.docx]

**Supplementary table 1: After excluding CNS neoplastic disease**

| **Biomarkers** | ***n* (per group)** | ***p*-values** | **AD with epilepsy** | **AD without epilepsy** |
| --- | --- | --- | --- | --- |
|  |  |  | **median, min-max** | **median, min-max** |
| NfL | 226 | 0.393 | 1365 (250-11800) | 1480 (270-52400) |
| GFAP | 83 | 0.719 | 810 (120-2610) | 800 (110-4930) |
| T-tau | 384 | 0.0021 | 620 (107-6940) | 567.5 (79-2480) |
| P-Tau | 364 | 0.0003 | 81 (18-253) | 73 (13-197) |
| Aβ42 | 364 | 0.0002 | 380 (144-1550) | 437 (140-1140) |
